# Ejaculate Collection Influences the Salivary Oxytocin Concentrations in Breeding Male Pigs

**DOI:** 10.3390/ani10081268

**Published:** 2020-07-25

**Authors:** Marina López-Arjona, Lorena Padilla, Jordi Roca, José Joaquín Cerón, Silvia Martínez-Subiela

**Affiliations:** 1 Interdisciplinary Laboratory of Clinical Analysis of the University of Murcia (Interlab-UMU), Regional Campus of International Excellence ‘Campus Mare Nostrum’, University of Murcia, Campus de Espinardo s/n, 30100 Espinardo, Murcia, Spain; marina.lopez10@um.es (M.L.-A.); silviams@um.es (S.M.-S.); 2 Department of Medicine and Animal Surgery, Veterinary Science, University of Murcia, 30100 Murcia, Spain; lorenaconcepcion.padilla@um.es (L.P.); roca@um.es (J.R.); 3 IMIB-Arrixaca, Regional Campus of International Excellence ‘Campus Mare Nostrum’, University of Murcia, 30100 Murcia, Spain

**Keywords:** oxytocin, boar, saliva, ejaculation

## Abstract

**Simple Summary:**

This study aimed to evaluate how the process of ejaculate collection affects oxytocin concentrations in saliva of boars used in artificial insemination. Saliva samples of 33 boars were collected the day before ejaculate collection, during the ejaculation time, and two hours after ejaculate collection. Free oxytocin and oxytocin linked to proteins were quantified in these saliva samples. Oxytocin concentrations during the ejaculation time were higher than the day before with oxytocin linked to proteins showing higher differences. In addition, younger boars, boars with higher libido intensity and boars of the Pietrain breed showed higher values of oxytocin in saliva during ejaculation than the day before. This study demonstrated that ejaculation influences the salivary oxytocin concentrations boars.

**Abstract:**

The objective of the present study was to evaluate the possible changes of oxytocin concentrations in saliva during and after ejaculate collection in breeding boars usually used in artificial insemination programs. Saliva samples of 33 boars were collected the day before ejaculate collection (DB), during the ejaculation time (T0) and two hours after ejaculate collection (T2). Free oxytocin and oxytocin linked to proteins concentrations were measured by two methods previously developed and validated for saliva of pigs. Younger boars, boars with higher libido intensity and boars of the Pietrain breed showed higher values of oxytocin in saliva during ejaculation than the day before. In addition, boars with higher libido showed higher concentrations two hours after ejaculate collection than during the day before. These changes were of higher magnitude and significance when oxytocin linked to proteins was measured. In conclusion, this study demonstrated for the first time that ejaculation influences the salivary oxytocin concentrations in breeding boars, although this influence varies according to age, libido and breed.

## 1. Introduction

Oxytocin is a neuropeptide hormone that is synthesized in the supraoptic and paraventricular nucleus of the hypothalamus [1]. This hormone has an important role in some physiological functions, such as labor and lactation [2]. In addition, oxytocin inhibits the secretion of glucocorticoids, the hormones associated with anxiety and stress [3]. Moreover, it is increasingly studied in the field of positive emotions and welfare in humans [4,5] and animals [6].

Oxytocin plays an important facilitating role during both male and female reproductive behavior in mammals [7,8]. In male reproduction, oxytocin is thought to be associated with ejaculation by increasing sperm number and contracting smooth muscle of reproductive ducts [9]. Intravenous or intraperitoneal injection of oxytocin contributes to ejaculation in different species, such as rats, rabbits and bulls [10,11,12]. Similarly, intra-testicular injection of oxytocin increased basal testosterone level in mice [13]. The addition of oxytocin to boar semen artificial insemination (AI) doses improves both sperm transport to the oviduct and conception rates [14,15,16], but it has no influence on the quality of sperm [15]. In humans, there is an increase in plasma oxytocin levels during, and at 5 min, after ejaculation [17,18].

In pigs, the use of saliva samples for laboratory analysis is a suitable non-invasive and non-stressful alternative to blood. Oxytocin can be measured in pig saliva, it changes throughout lactation period in nursing sows [19] and it is associated to some behaviours [20]. In addition, oxytocin in saliva samples increases in men after sexual self-stimulation [21]. However, to the best of our knowledge, there are no studies about possible changes of oxytocin in saliva related to the ejaculation process in boars. It could be postulated that oxytocin could increase during the ejaculate collection time, possibly due to two main reasons: (1) the role of the oxytocin in the ejaculation, increasing contractility of reproductive smooth muscle [22], and (2) the possible emotional status associated with ejaculation [23].

The purpose of this study was to evaluate possible changes in the salivary oxytocin concentrations during ejaculate collection time in breeding boars. In addition, the influence of breed, age and libido of breeding boars in salivary oxytocin levels was also be evaluated. To accomplish these goals, two new methods previously developed and validated for oxytocin measurement in pig saliva were used, one based in the use of a monoclonal antibody that measures free oxytocin [19], and the other using a polyclonal antibody that measures oxytocin linked to protein [24].

## 2. Materials and Methods

### 2.1. Animals and Ejaculate Collection

A total of 33 mature and fertile breeding boars of three different breeds (2 Landrace, 13 Duroc and 18 Pietrain boars) were included in the study. Boars were housed in a farm located in southern Spain. The boars were housed in individual pens in a building with a controlled environment (16 h of light per day and 18–24 °C), with free access to water and fed with commercial feedstuff twice a day. The boars were included in artificial insemination (AI) programs with a regular ejaculate collection of two ejaculates per week. For ejaculate collection, the boars were moved from their pens to another individual pen containing a dummy sow. Once free mounted to the dummy, entire ejaculate was collected using the semi-automatic procedure called Collectis^®^ (IMV technologies, L’Aigle, France).

### 2.2. Saliva Collection and Oxytocin Measurement

Saliva samples were collected using Salivette tubes (Sarstedt, Aktiengesellschaft & Co. D−51588 Nümbrecht, Germany) that contained a sponge. For sampling, the boars chewed the sponge, which was clipped to a metal rod, until the sponge was moist. The sponges were placed in test Salivette tubes and refrigerated until arrival at the laboratory. The tubes were then centrifuged at 3500 rpm at 4 °C for 10 min. Finally, saliva samples were stored at −80 °C until analysis. The research protocols were approved by the Bioethical Commission of Murcia University according to the European Council Directives regarding the protection of animals used for experimental purposes (approval number, 235/2016; approval date, 25/04/2016).

### 2.3. Measurement of Salivary Oxytocin Concentration

Saliva samples were thawed at room temperature and oxytocin concentrations were measured using two different methods previously developed and validated for pig saliva [19,24]. One method was a direct competition assay based on AlphaLISA (PerkinElmer Inc., Waltham, MA, USA) technology using monoclonal antibody against oxytocin, while the other consisted of an indirect competition assay based on AlphaLISA technology using polyclonal antibody against oxytocin. Although these assays have been previously described [19,24], in brief, AlphaLISA monoclonal method was performed as follows: 15 µL of sample diluted 1:2 with AlphaLISA buffer and 15 µL of acceptor beads were added to the well and after 90 min, 10 µL of biotinylated oxytocin was added and incubated for 60 min. Then, 10 µL of donor beads was added and after 30 min in the dark, the fluorescence intensity was measured using the Enspire Multimode Plate Reader (PerkinElmer Inc.). In the case of the AlphaLISA polyclonal method, 10 µL of sample diluted 1:2 with AlphaLISA buffer and 10 µL of anti-oxytocin polyclonal antibody were added to the well and after 45 min, 10 µL of protein G acceptor beads was added and incubated for 45 min. Then, 10 µL of biotinylated oxytocin was added and after 45 min, 10 µL of donor beads was added and incubated in the dark for 30 min. Subsequently, the fluorescence intensity was measured using the Enspire Multimode Plate Reader (Perkin Elmer Inc., USA). The accuracy of assays had a correlation coefficient which ranged between 0.96 and 0.99 in the case of the monoclonal method and between 0.98 and 0.99 in the case of the polyclonal method. The intra-assay coefficients of variation were between 5.02–6.07% in the case of the monoclonal method and 4.36–11.37% in the case of the polyclonal method, while the inter-assay coefficients of variation were between 4.60–17.70% in the case of the monoclonal method and 8.52–13.38% in the case of the polyclonal method. The sensitivity of the assays was 112.95 pg/mL and 58.40 pg/mL in the cases of the monoclonal and polyclonal method, respectively.

### 2.4. Experimental Design

Saliva samples from the 33 boars were collected at three different times in each boar: the day before ejaculate collection (DB); immediately after starting the ejaculation (T0) and two hours after ejaculation (T2). This collection protocol was carried out in February, 2019. Libido was measured according to a three-point scale adapted from that of Kozink et al. [25], namely a value of 1 for boars that showed little interest in the dummy sow and took more than 10 min to mount it; a value of 2 for the boars that did not show much interest but mounted it in less than 10 min; and a value of 3 for the boars that interacted with the dummy and quickly mounted it.

### 2.5. Statistical Analysis

Medians and 25th–75th percentiles were calculated by use of routine descriptive statistical procedures and computer software (Excel 2016, Microsoft Corporation, Redmond, WA, USA). Statistical analyses were performed using Graph Pad Software Inc (GraphPad Prism, version 5 for Windows, Graph Pad Software Inc, San Diego, CA, USA). The Shapiro–Wilk test was performed to evaluate the data distribution, which did not follow a normal distribution. Therefore, data were log-transformed. One-way repeated measures ANOVA followed by uncorrected Fisher’s LSD were used to compare oxytocin values obtained at the different times, namely DB, T0 and T2. Spearman’s correlation coefficient was calculated between the analytical parameters with oxytocin concentrations measured with AlphaLISA monoclonal and polyclonal method. The strength of the correlation was assessed by the Rule of Thumb [26], according to which an R value between 0.90 to 1 was considered to have very high correlation, 0.70 to 0.90 high correlation, 0.50 to 0.70 moderate correlation, 0.30 to 0.50 low correlation and less than 0.30 little, if any, correlation. Results were reported as median and 25th−75th percentiles (in text) and line-box plots (in Figures) and a *p* < 0.05 was considered significant.

## 3. Results

The salivary oxytocin concentrations measured with monoclonal and polyclonal assays showed a low correlation (R = 0.323, *p* < 0.05). As such, the results for each assay are presented separately.

### 3.1. Oxytocin Concentrations Measured with the Monoclonal Assay

**Figure 1:**
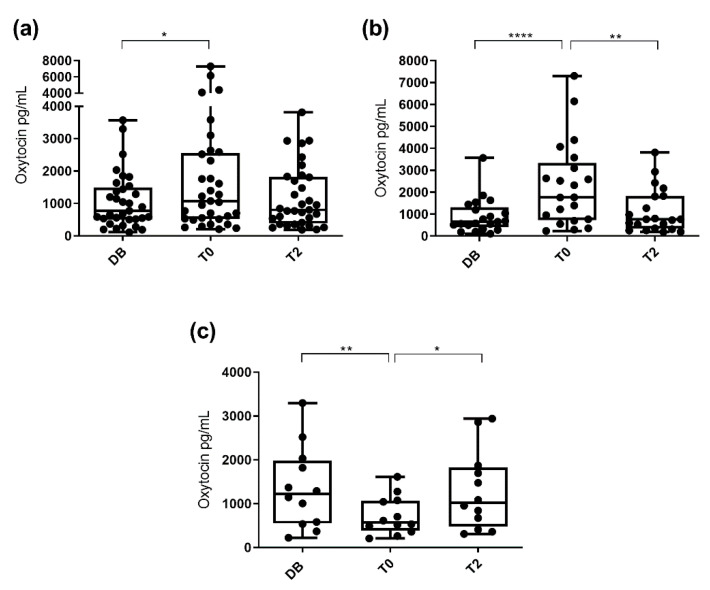
Changes in salivary oxytocin concentrations measured with AlphaLISA monoclonal assay at different times: the day before ejaculate collection (DB), immediately after starting the ejaculation (T0) and two hours after ejaculation (T2). Data for all 33 boars (**a**), data for the 21 boars showing increased oxytocin concentration at ejaculation time (**b**) and data for the 12 boars that did not show increased oxytocin concentrations at ejaculation time (**c**). The plots show medians (line within box), 25th and 75th percentiles (boxes), min and max values (whiskers) and individual data points. Asterisks indicate differences between times (**** *p* ≤ 0.0001; ** *p* ≤ 0.01; * *p* ≤ 0.05).

Salivary oxytocin concentrations were significantly higher (*p* < 0.05) at T0 (1077.0 pg/mL; 25−75th percentile: 527.3–2555.0 pg/mL) than at DB time (775.6 pg/mL; 25–75th percentile: 512.8–1494.0 pg/mL) but there were no significant differences (*p* > 0.05) when compared with T2 (802.6 pg/mL; 25–75th percentile: 386.5–1821.0 pg/mL). Interestingly, not all boars showed the same pattern of variation. While some boars showed increased concentrations of oxytocin at T0 compared to DB, other boars showed the opposite behavior (decreased concentrations at T0). Therefore, changes in salivary oxytocin concentrations were evaluated separately in each of the two boar groups. Oxytocin concentrations in the group that showed an increase at T0 (*n* = 21) were higher (*p* < 0.01) at T0 (1767.0 pg/mL; 25–75th percentile: 737.7–3342.0 pg/mL) than at DB (653.4 pg/mL; 25–75th percentile: 413.3–1322.0 pg/mL) and T2 (771.2 pg/mL; 25–75th percentile: 336.5–1821.0 pg/mL). Oxytocin concentrations in the other group, which showed a decrease at T0 (*n* = 12), were lower (*p* < 0.05) at T0 (574.8 pg/mL; 25–75th percentile: 393.2–1069.0 pg/mL) than at DB (1219.0 pg/mL; 25–75th percentile: 550.6–1981.0 pg/mL) and T2 (1022.0 pg/mL; 25–75th percentile: 477.2–1829.0 pg/mL). These results are shown in Figure 1.

**Figure 2:**
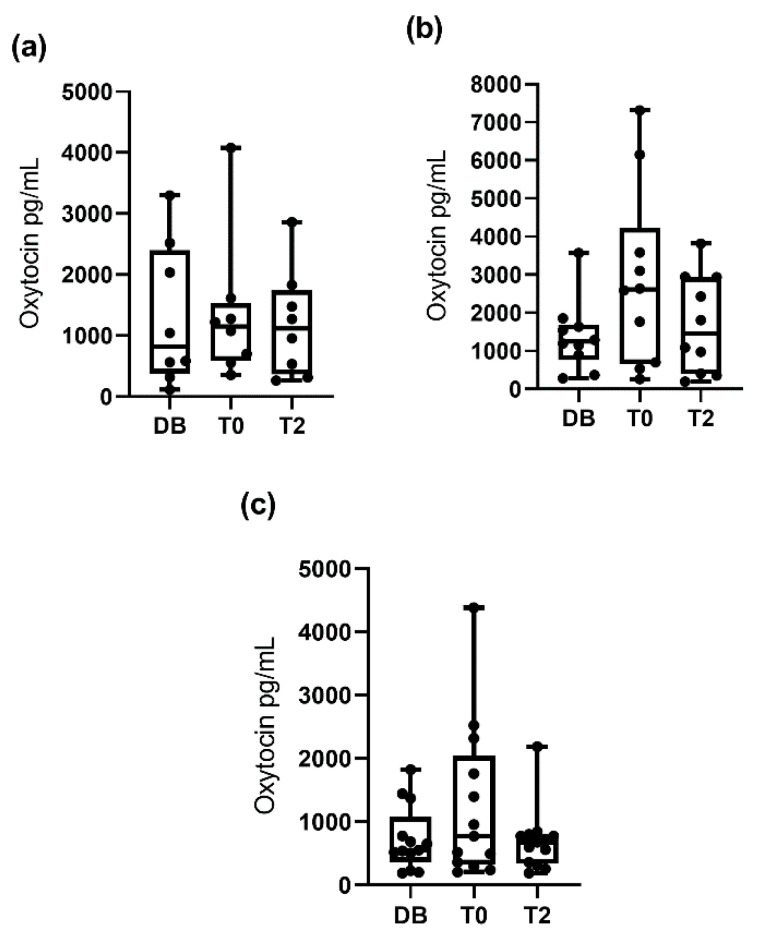
Changes in salivary oxytocin concentrations measured with AlphaLISA monoclonal assay at different times: the day before ejaculate collection (DB), immediately after starting the ejaculation (T0) and two hours after ejaculation (T2). Figure (**a**) shows the data for boars aged 12–24 months and (**b**,**c**) those for boars aged 24–36 and aged more than 36 months, respectively. The plots show medians (line within box), 25th and 75th percentiles (boxes), min and max values (whiskers) and individual data points.

**Figure 3:**
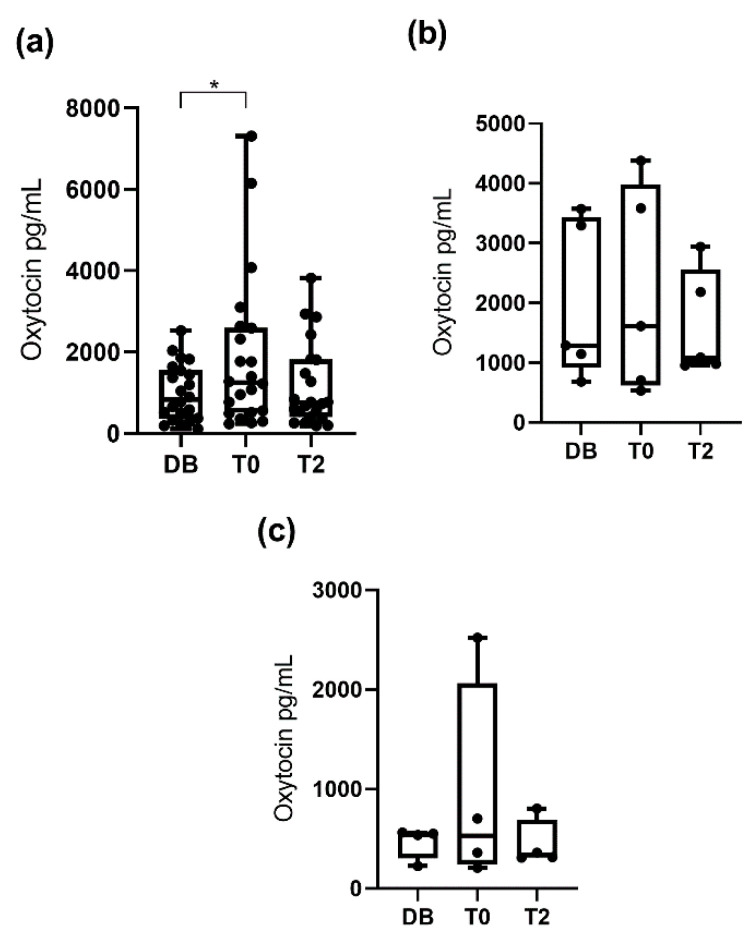
Changes in salivary oxytocin concentrations measured with AlphaLISA monoclonal assay at different times: the day before ejaculate collection (DB), immediately after starting the ejaculation (T0) and two hours after ejaculation (T2). Figure (**a**) shows the data for boars with libido intensity of 3 and (**b**,**c**) those with libido intensity of 2 and 1, respectively. The plots show medians (line within box), 25th and 75th percentiles (boxes), min and max values (whiskers) and individual data points. Asterisk indicate differences between times (* *p* ≤ 0.05).

When pigs were classified by age (boars aged 12 to 24 months, aged 24 to 36 months, and aged more than 36 months), no significant differences in salivary oxytocin between times were seen within each group (Figure 2). However, when the pigs were classified according to their libido, boars with a libido intensity of 3 had higher (*p* < 0.05) oxytocin concentrations at T0 (1250.0 pg/mL; 25–75th percentile: 511.9–2599.0 pg/mL) than DB (834.9 pg/mL; 25–75th percentile: 357.8–1565.0 pg/mL) and did not show significant changes at T2 (749.5 pg/mL; 25–75th percentile: 397.6–1815.0 pg/mL), whereas boars with libido intensity values of 1 and 2 showed similar salivary oxytocin concentrations across the three collection times (Figure 3).

The frequency of individuals having increases in oxytocin concentrations at T0 was different depending on the breed. It was found that around 83.4% of Pietrain boars showed higher oxytocin concentrations at T0 than at DB (*p* < 0.01) and T2 (*p* < 0.05). However, only 46.1% of Duroc boars showed higher oxytocin concentrations at T0 but without differences between DB and T0. The two Landrace boars did not show significant changes at the different times.

### 3.2. Oxytocin Concentration Measured with the Polyclonal Assay

**Figure 4:**
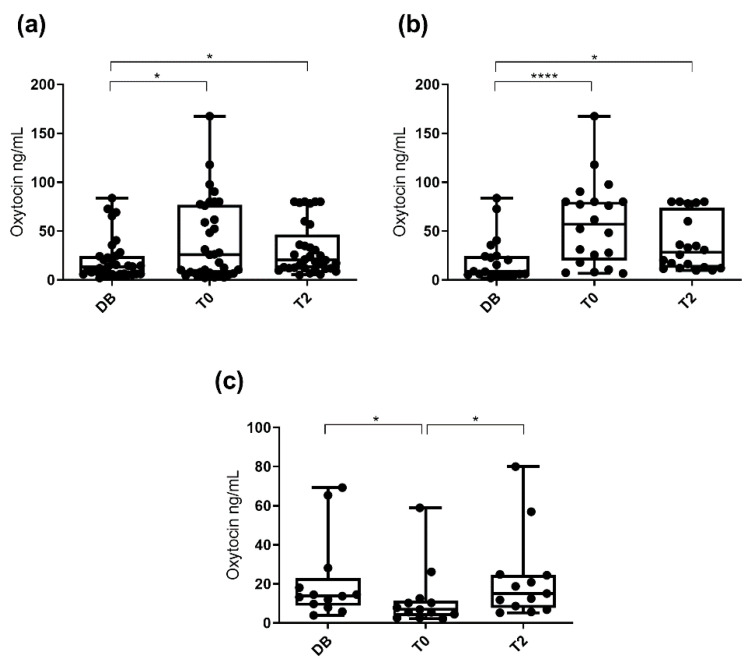
Changes in salivary oxytocin concentrations measured with AlphaLISA polyclonal assay at different times: the day before ejaculate collection (DB), immediately after starting the ejaculation (T0) and two hours after ejaculation (T2). Data for all 33 boars (**a**), data for the 20 boars showing increased oxytocin concentration at ejaculation time (**b**), and data for the 13 boars that did not show increased oxytocin concentrations at ejaculation time (**c**). The plots show medians (line within box), 25th and 75th percentiles (boxes), min and max values (whiskers) and individual data points. Asterisks indicate differences between times (**** *p* ≤ 0.0001; * *p* ≤ 0.05).

Salivary oxytocin concentrations were significantly higher (*p* < 0.05) at T0 (25.8 ng/mL; 25–75th percentile: 7.2–76.7 ng/mL) and T2 (20.3 ng/mL; 25–75th percentile: 11.9–46.5 ng/mL) than at DB time (13.3 ng/mL; 25–75th percentile: 6.1–24.5 ng/mL). Regarding the different pattern of variation, changes in oxytocin concentrations were evaluated separately in each of the two boar groups depending on the increase or decrease at T0. Oxytocin concentrations in the group that showed an increase at T0 (*n* = 20) were higher (*p* < 0.05) at T0 (57.2 ng/mL; 25–75th percentile: 19.9–80.0 ng/mL) and T2 (28.4 ng/mL; 25–75th percentile: 12.4–73.7 ng/mL) than DB (8.7 ng/mL; 25–75th percentile: 5.8–24.8 ng/mL). Oxytocin concentrations in the group that showed a decrease at T0 (*n* = 13) were higher (*p* < 0.05) at DB (13.8 ng/mL; 25–75th percentile: 8.8–23.2 ng/mL) and T2 (15.1 ng/mL; 25–75th percentile: 7.8–24.7 ng/mL) than T0 (6.9 ng/mL; 25–75th percentile: 3.6–11.5 ng/mL). These results are shown in Figure 4.

**Figure 5:**
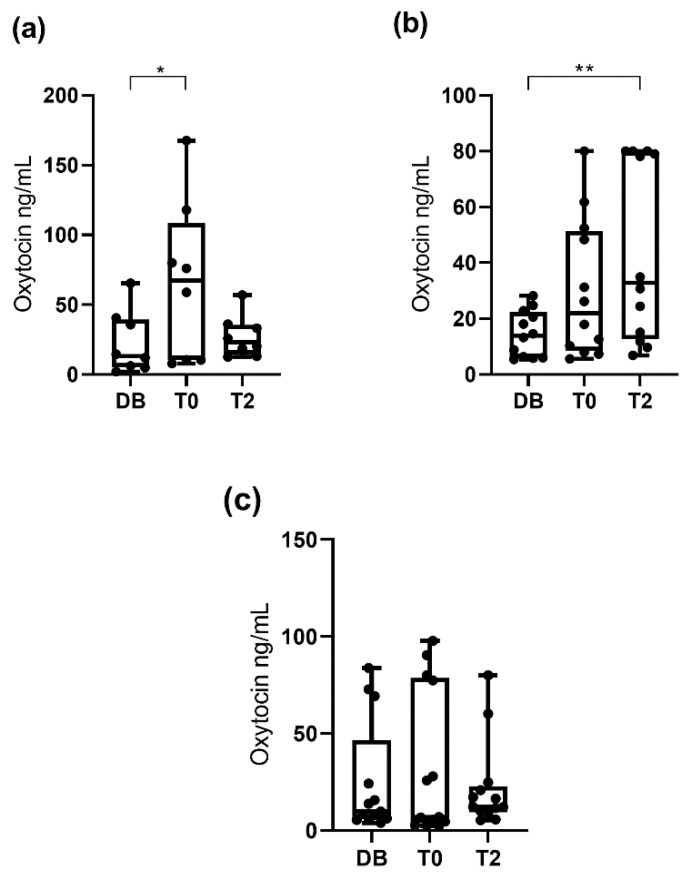
Changes in salivary oxytocin concentrations measured with AlphaLISA polyclonal assay at different times: the day before ejaculate collection (DB), immediately after starting the ejaculation (T0), and two hours after ejaculation (T2). Figure (**a**) shows the data for boars aged 12–24 months and (**b**) and (**c**) those for boars aged 24–36 and aged more than 36 months, respectively. The plots show medians (line within box), 25th and 75th percentiles (boxes), min and max values (whiskers) and individual data points. Asterisks indicate differences between times (** *p* ≤ 0.01; * *p* ≤ 0.05).

**Figure 6:**
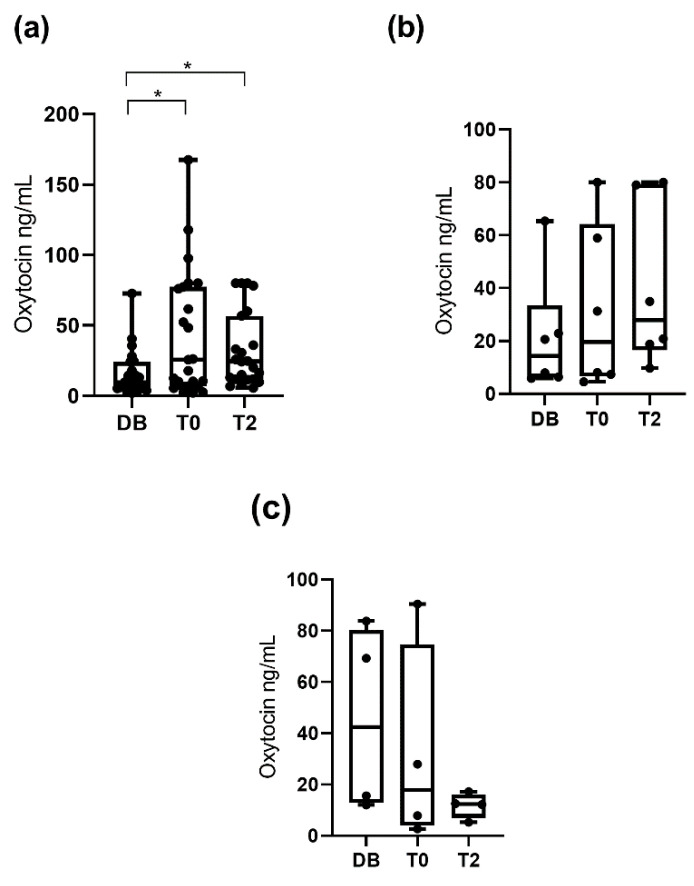
Changes in salivary oxytocin concentrations measured with AlphaLISA polyclonal method at different times: the day before ejaculate collection (DB), immediately after starting the ejaculation (T0) and two hours after ejaculation (T2). Figure (**a**) shows the data for boars with libido intensity of 3 and (**b**,**c**) those with libido intensity of 2 and 1, respectively. The plots show medians (line within box), 25th and 75th percentiles (boxes), min and max values (whiskers) and individual data points. Asterisk indicate differences between times (* *p* ≤ 0.05).

When pigs were classified by age (boars aged 12 to 24 months, aged 24 to 36 months, and aged more than 36 months), significant differences between times were seen in boars aged 12 to 24 months and in boars aged 24 to 36 months (Figure 5). Oxytocin concentrations observed in boars aged 12 to 24 months were higher (*p* < 0.05) at T0 (67.5 ng/mL; 25–75th percentile: 10.4–108.4 ng/mL) than at DB (13.3 ng/mL; 25–75th percentile: 5.3–39.3 ng/mL). Oxytocin concentrations in boars aged 24 to 36 months were higher (*p* < 0.01) at T2 (32.9 ng/mL; 25–75th percentile: 12.7–79.7 ng/mL) than DB (13.9 ng/mL; 25–75th percentile: 6.1–22.3 ng/mL). Boars aged more than 36 months showed similar salivary oxytocin concentrations at the three collection times. When the pigs were classified according to their libido, boars with a libido intensity of 3 showed higher (*p* < 0.05) oxytocin concentrations at T0 (25.8 ng/mL; 25–75th percentile: 6.9–77.4 ng/mL) and at T2 (24.5 ng/mL; 25–75th percentile: 11.9–56.9 ng/mL) than DB (9.8 ng/mL; 25–75th percentile: 5.9–24.2 ng/mL). Boars with libido intensity 2 and 1 showed similar salivary oxytocin concentrations at the three collection times (Figure 6).

The frequency of individuals having increased oxytocin concentrations at T0 was different depending on the breed. We found that 72.2% of Pietrain boars showed higher oxytocin concentrations at T0 than at DB (*p* < 0.01). However, 46.1% of Duroc boars showed an increase at T0 but without differences between DB and T0, although oxytocin concentrations at T2 were higher (*p* < 0.05) than at DB. The two Landrace boars did not show significant changes at the different times.

## 4. Discussion

This manuscript reports, for the first time, changes in oxytocin concentrations in saliva associated with the ejaculation process in a livestock species. To the best of our knowledge, increases in oxytocin levels after ejaculation have only been reported in cerebrospinal fluid of rats [27], in blood of rabbits [28] and blood and saliva of humans [17,18,21]. All these studies reported an increase of oxytocin levels associated with ejaculation and support our hypothesis that oxytocin would also increase in pig saliva.

Specific studies addressing how oxytocin reaches saliva in pigs have not been reported. However, mammals share essential oxytocin system characteristics, such as production of oxytocin in the hypothalamus and peripheral and central release [29]. Oxytocin is synthesized in the hypothalamic supraoptic and paraventricular nuclei as a neurohormone and is released through the posterior pituitary gland into the bloodstream [30]. In humans, oxytocin reaches the salivary glands via blood circulation through active transport mechanisms [31]. However, the dynamic of oxytocin in both fluids (blood and saliva) could be different, since saliva concentrations of oxytocin do not reflect peripherally circulating oxytocin after exogenous administration in humans [32]. Martin et al. [33] found that oxytocin concentrations in human saliva are more correlated with those in cerebrospinal fluid, and therefore with central oxytocin, than with those of blood plasma.

In this study, two assays were used for measurement of salivary oxytocin, namely monoclonal and polyclonal assays. The results evidenced that the polyclonal assay detects changes of higher magnitude in salivary oxytocin concentrations, especially at 2 h after ejaculate collection. This could be related to the ability of polyclonal assays to detect different metabolites of the oxytocin molecule together with oxytocin bound to other proteins [24] that could remain 2 h after ejaculation. Differences between both assays have also been described in a previous study measuring oxytocin in lactating sows [24]. Together, these results would make polyclonal assays the assay of choice for measuring changes in salivary oxytocin in breeding boars. Accordingly, the discussion will be focused on the results achieved using the polyclonal assay.

The increased salivary oxytocin concentration during ejaculation found in our study reflect its involvement in the ejaculation process [9,34]. Oxytocin contributes to the modulation of sexual behavior [35] and is also involved in the control of male fertility, promoting the transport of sperm through the regulation of basal contractility of smooth muscle in the cauda epididymis [34], as well as stimulation of contraction of the prostate during ejaculation [36]. In addition to its physiological role in the mechanisms related to ejaculation, the increase in oxytocin during ejaculate collection time could also be associated with positive emotions experienced by boars during ejaculation. Oxytocin may represent a link between the social and physical feelings and emotions and sexual behavior [5,37], being released through sensory stimuli, such as touch, warmth and odor [20,38,39]. In addition, oxytocin has become a central component of the mechanisms mediating the well-being and anti-stress effects of positive social interactions in humans [40,41] and animals [6,42].

Interestingly, not all boars showed increased oxytocin concentrations during ejaculation time, as it increased in only 20 of the 33 breeding boars under study. There are several factors that can influence the sexual behavior of boars at ejaculation, such as genetic factors [43]. In this study, more Pietrain than Duroc boars showed increased oxytocin concentrations at T0. The weaker libido of Duroc boars compared with those of others breeds [44,45], as well as the lower total number of sperm and percentage of live sperm in the ejaculates compared with those of Pietrain boars [46], could explain the lower or non-existent increase in salivary oxytocin concentrations at ejaculation time. Furthermore, the age of boars is another influencing factor, as younger boars showed the highest salivary oxytocin concentrations during the ejaculate collection, while the older boars did not show changes between times. We could not find information in the literature to explain differences in salivary oxytocin levels related to age. However, previous studies in boars found that those aged 18 to 24 months had a higher percentage of motile sperm than those aged more than 30 months, which also had higher percentages of sperm abnormalities, such as simple bent tails [47]. In line with this, pregnant women of the first child showed higher serum oxytocin levels compared to pregnant women who already had children [48].

Libido was another factor influencing the salivary oxytocin levels. Boars with high libido showed the highest oxytocin concentrations in saliva at ejaculation time, while boars with moderate or low libido did not show changes in oxytocin concentrations between times. Previous studies in humans found that blood oxytocin was positively related with intensity of sexual behavior and couple interaction [49]. Therefore, it could be postulated that increased libido in boars could be caused by a higher oxytocin synthesis. However, it could also be to the contrary, i.e., increased libido induces increased synthesis of oxytocin. More studies are needed to clarify this issue.

## 5. Conclusions

In conclusion, this study demonstrated, for the first time, changes in salivary oxytocin concentration in breeding boars during ejaculate collection time. However, the magnitude and significance of changes are different depending on the assay used for its measurement, with the polyclonal assay better suited for detecting changes in oxytocin concentrations than the monoclonal assay. In addition, increases in salivary oxytocin concentrations at ejaculation time in younger boars, in boars showing greater libido intensity, and those of the Pietrain breed, were observed. Overall, this study opens a new line of research about the possible use of salivary oxytocin concentration as potential biomarker of sexual behavior of breeding male pigs. In this context, it would also be interesting to evaluate whether the salivary levels of oxytocin are related to the quantity and quality of semen. This study also suggests the potential use of salivary levels of oxytocin for evaluating the welfare of boars, and for detecting or monitoring problems associated with a low reproductive performance.
